# MCU-induced mitochondrial calcium uptake promotes mitochondrial biogenesis and colorectal cancer growth

**DOI:** 10.1038/s41392-020-0155-5

**Published:** 2020-05-05

**Authors:** Yang Liu, Mingpeng Jin, Yaya Wang, Jianjun Zhu, Rui Tan, Jing Zhao, Xiaoying Ji, Chao Jin, Yongfeng Jia, Tingting Ren, Jinliang Xing

**Affiliations:** 1 State Key Laboratory of Cancer Biology and Department of Physiology and Pathophysiology, Fourth Military Medical University, Xi’an, China; 2 Department of Pathology, Basic Medical College, Inner Mongolia Medical University, Huhhot, China; 3 College of Chemistry and Chemical Engineering, Xi’an University of Science and Technology, Xi’an, China; 4 Department of Cell Biology and Genetics, Basic Medicine College, Shanxi Medical University, Taiyuan, China; 5 Department of Orthopedics, Xijing Hospital, Fourth Military Medical University, Xi’an, China; 6 Department of General Surgery, Tangdu Hospital, Fourt h Military Medical University, Xi’an, China; 7 State Key Laboratory of Cancer Biology and Experimental Teaching Center of Basic Medicine, Fourth Military Medical University, Xi’an, China

**Keywords:** Cancer therapy, Oncogenes

## Abstract

Mitochondrial calcium uniporter (MCU) has an important role in regulating mitochondrial calcium (Ca^2+^) homeostasis. Dysregulation of mitochondrial Ca^2+^ homeostasis has been implicated in various cancers. However, it remains unclear whether MCU regulates mitochondrial Ca^2+^ uptake to promote cell growth in colorectal cancer (CRC). Therefore, in the present study the expression of MCU in CRC tissues and its clinical significance were examined. Following which, the biological function of MCU-mediated mitochondrial Ca^2+^ uptake in CRC cell growth and the underlying mechanisms were systematically evaluated using in in vitro and in vivo assays, which included western blotting, cell viability and apoptosis assays, as well as xenograft nude mice models. Our results demonstrated that MCU was markedly upregulated in CRC tissues at both the mRNA and protein levels. Upregulated MCU was associated with poor prognosis in patients with CRC. Our data reported that upregulation of MCU enhanced the mitochondrial Ca^2+^ uptake to promote mitochondrial biogenesis, which in turn facilitated CRC cell growth in vitro and in vivo. In terms of the underlying mechanism, it was identified that MCU-mediated mitochondrial Ca^2+^ uptake inhibited the phosphorylation of transcription factor A, mitochondrial (TFAM), and thus enhanced its stability to promote mitochondrial biogenesis. Furthermore, our data indicated that increased mitochondrial Ca^2+^ uptake led to increased mitochondrial production of ROS via the upregulation of mitochondrial biogenesis, which subsequently activated NF-κB signaling to accelerate CRC growth. In conclusion, the results indicated that MCU-induced mitochondrial Ca^2+^ uptake promotes mitochondrial biogenesis by suppressing phosphorylation of TFAM, thus contributing to CRC cell growth. Our findings reveal a novel mechanism underlying mitochondrial Ca^2+^-mediated CRC cell growth and may provide a potential pharmacological target for CRC treatment.

## Introduction

Colorectal cancer (CRC) represents a huge public health burden worldwide and has higher rates of incidence in developed countries.^1^ Every year, CRC leads to the death of nearly 700,000 individuals, making it one of the most deadly cancers.^1^ Although there has been progress in the early diagnosis and treatment of CRC, the mechanism underlying the pathogenesis of CRC remains to be elucidated. Thus, studies that explore the molecular mechanisms contributing to the growth of CRC cells are urgently needed in order to develop novel therapeutic strategies.

Intracellular calcium (Ca^2+^), which is a ubiquitous second messenger, plays important roles in various types of biological events. Owing to the significance of Ca^2+^ in signaling pathways, the level of Ca^2+^ in cells is strictly controlled. Altered Ca^2+^ homeostasis may lead to different pathological conditions, depending on the type of cell involved.^2^ For instance, it has been well documented that Ca^2+^ signaling is a key regulator in a wide range of cellular processes, including tumor growth, progression, and metastasis.^3^ This demonstrates that dysregulated Ca^2+^ signaling is often detrimental and has been associated with each of the “cancer hallmarks.”^4^

Owing to its Ca^2+^ buffering capacity, the mitochondrion is an important organelle responsible for maintaining intracellular Ca^2+^ homeostasis. Ca^2+^ influx into mitochondria, which is primarily regulated by the mitochondrial calcium uniporter (MCU) complex, is a pleiotropic signal that controls a broad spectrum of cellular functions, including vital metabolic pathways, production of reactive oxygen species (ROS), and the life/death decisions of cells.^5^ The understanding of the MCU complex has rapidly increased due to a myriad of recent studies that have identified the pore-forming molecule MCU and its regulatory subunits, including essential MCU regulator (EMRE), MCU regulator 1 (MCUR1), MCU-dominant-negative β-subunit (MCUb), mitochondrial calcium uptake (MICU) 1, MICU2, and MICU3.^6^ Abnormal changes in the expression levels or functional role of one or more members of the MCU complex have been associated with cancer-related phenotypes in different types of cancers, such as hepatocellular carcinoma, breast cancer, colon cancer, and pancreatic cancer.^7^

In recent years, an increasing number of studies are beginning to pay close attention to the functional role of MCU, a key component in the MCU complex, in different diseases, especially in cancers. Growing evidence has demonstrated that MCU possesses pivotal roles in different types of cancers.^8–10^ For example, it has been reported that the expression of MCU elevated in basal-like and estrogen receptor-negative breast cancers, and the depletion of MCU promotes caspase-independent apoptosis in breast cancer cells.^9^ Similarly, our previous study demonstrated that MCU is upregulated in HCC cells and promotes HCC cell survival via the ROS/AKT/MDM2 pathway.^11^ Furthermore, Tosatto et al.^12^ have reported that MCU is instrumental for the growth of triple-negative breast cancer. One recent study also indicated that high-mitochondrial Ca^2+^ mediated by MCU increases prostate cancer cell proliferation by inhibiting mitochondrial permeability transition pore (mPTP).^13^ Although the biological role of MCU in the progression of several cancer types has been extensively studied, it remains unclear whether MCU is involved in CRC cell growth via the regulation of mitochondrial Ca^2+^ uptake.

To further investigate the potential role of MCU and mitochondrial Ca^2+^ in CRC growth, we investigated the expression level of MCU and the biological role of MCU-mediated mitochondrial Ca^2+^ homeostasis in CRC cell growth. To the best of our knowledge, this is the first study to demonstrate the functional significance of MCU-mediated mitochondrial Ca^2+^ homeostasis in CRC and reveal a novel underlying mechanism, thus providing a potential therapeutic strategy for patients with CRC.

## Results

### Upregulation of MCU is associated with poor prognosis in patients with CRC

**Fig. 1:**
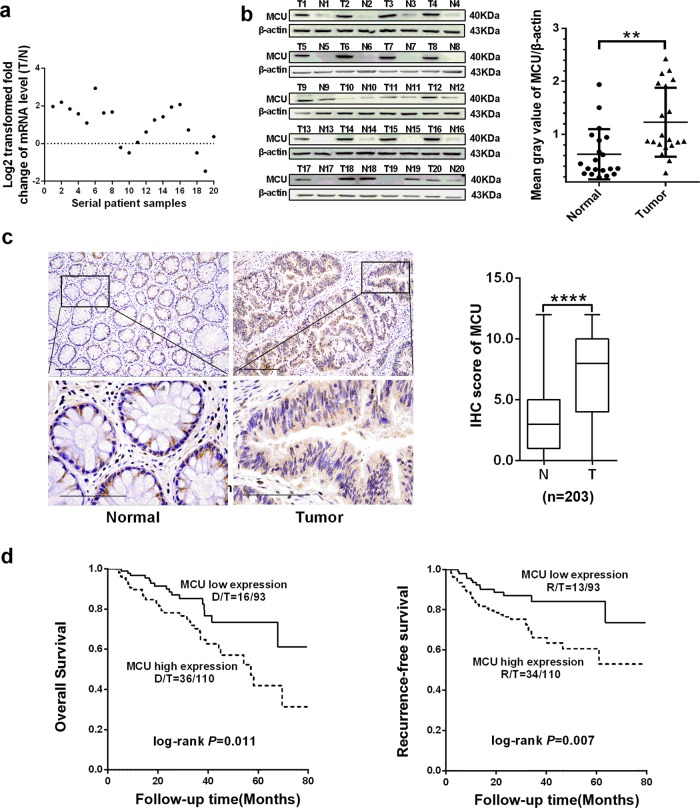
Upregulated MCU is associated with poor prognosis in patients with CRC. **a** Reverse transcription-quantitative PCR was performed to measure the relative mRNA expression of MCU in 20 paired fresh tissues. The ratio of relative mRNA expression between tumor and normal tissues was log2-transformed. **b** Western blotting analysis to measure protein expression level of MCU in 20 paired fresh tissues. Mean gray values of MCU and β-actin (internal control) expression were determined by Quantity One software. **c** Representative IHC staining images (Left) and IHC score (Right) of MCU in 203 paired tissues, which includes the aforementioned 20 paired tissues. **d** Kaplan–Meier plot of overall and recurrence-free survival of patients with CRC depending on MCU expression. *P < 0.05; **P < 0.01. MCU mitochondrial calcium uniporter, CRC colorectal cancer, T tumor, N normal, IHC immunohistochemical

To determine the biological role of MCU in tumorigenesis of CRC, reverse transcription-quantitative PCR (RT-qPCR) and western blotting assays were performed to examine the expression level of MCU in 20 paired CRC and adjacent normal tissues. Our data revealed that MCU was markedly upregulated in the majority of CRC tissues at both the mRNA and protein levels compared with paired non-malignant tissues (Fig. 1a, b). In addition, we evaluated the expression level of other MCU complex subunits in human CRC tissues based on public RNA-seq data from The Cancer Genome Atlas (TCGA) database and the RT-qPCR analysis of our samples. TCGA data analysis from 41 paired samples showed that MICU1 was significantly downregulated at the mRNA level in CRC tissues compared with the adjacent tissues, while no significant differences were observed in the mRNA expression of MICU2, MCUb, MCUR1, and EMRE (Supplementary Fig. S1a). Furthermore, RT-qPCR analysis of 20 paired tissue samples showed similar results, indicating that MICU1 mRNA expression is downregulated in CRC tissues (Supplementary Fig. S1b).

Our result was further validated by immunohistochemical (IHC) analysis in 159 of 203 (78%) paired CRC and adjacent normal tissues, indicating that CRC tissues had a significantly higher median MCU IHC score compared with adjacent non-tumor tissues (*P* < 0.001, Fig. 1c). Moreover, MCU expression was stratified into two groups based on the median value of the IHC score. Kaplan–Meier analysis showed that patients with CRC that had a high expression of MCU, displayed notably shorter overall survival (OS) and recurrence-free survival (RFS) compared with those who had low MCU expression (log-rank *P* = 0.011 and 0.007, respectively) (Fig. 1d).

### Upregulation of MCU enhances the mitochondrial Ca^2+^ uptake in CRC cells

**Fig. 2:**
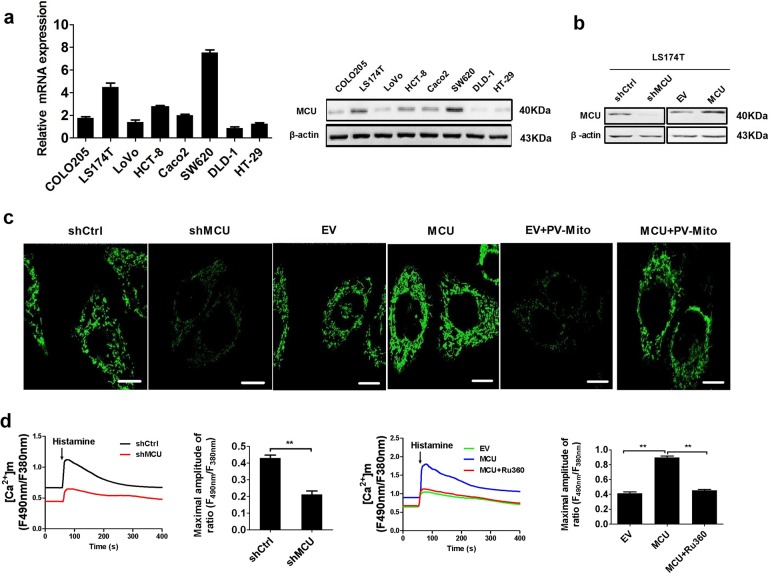
Upregulation of MCU increases mitochondrial Ca2+ uptake in CRC cells. **a** Reverse transcription-quantitative PCR and western blotting analyses for mRNA and protein expression levels of MCU in different CRC cell lines (COLO205, LS174T, LoVo, HCT-8, CaCo-2, SW620, DLD-1, and HT-29). **b** Western blotting analysis to measure MCU protein expression in LS174T cells, treated as indicated. **c** Confocal microscope images of [Ca^2+^]m using MitoPericam (Green) to label mitochondria in LS174T cells, treated as indicated. Scale bar, 5 µm. **d** Confocal microscope analysis of mitochondrial [Ca^2+^]m in CRC cells with a panel of treatment in response to 10 µM histamine. Ruthenium 360 (10 µM) was used to inhibit MCU activity. *P < 0.05; **P < 0.01. MCU mitochondrial calcium uniporter, CRC colorectal cancer, shCtrl control shRNA, shMCU shRNA against MCU, EV empty vector, [Ca^2+^]_m_ mitochondrial Ca^2+^ levels, PV-Mito expression vector encoding parvalbumin with mitochondria target sequence, Ca^2+^ calcium

As MCU has a critical role in maintaining mitochondrial Ca^2+^ homeostasis,^6^ we first determined whether MCU exerted an effect on the level of mitochondrial Ca^2+^ in LS174T and HCT-8 cells, which exhibited a median expression level of MCU (Fig. 2a). Furthermore, the overexpression and knockdown of MCU in LS174T and HCT-8 cells were confirmed by western blotting (Fig. 2b and Supplementary Fig. S2a). As shown in Fig. 2c, knockdown of MCU in LS174T cells resulted in a marked reduction in the basal level of mitochondrial Ca^2+^ ([Ca^2+^]_m_) compared with control cells, whereas MCU overexpression considerably increased basal [Ca^2+^]_m_. We also found that the cells treated with the [Ca^2+^]m buffering protein parvalbumin (PV) using PV-Mito showed decreased mitochondrial Ca^2+^ levels and thus reversed the MCU-induced mitochondrial Ca^2+^ uptake (Fig. 2c). Similar findings were obtained in HCT-8 cells (Supplementary Fig. S2b). Histamine, an InsP3-linked agonist, was employed to examine the effect of MCU on the ability of mitochondrial Ca^2+^ uptake by rapidly increasing the intracellular Ca^2+^ ([Ca^2+^]_c_), and subsequently promoting the mitochondrial Ca^2+^ uptake. The capability of mitochondrial Ca^2+^ uptake was suppressed in MCU-knockdown CRC cells, whereas overexpression of MCU in CRC cells increased mitochondrial Ca^2+^ uptake (Fig. 2d). Similar findings were obtained in HCT-8 cells (Supplementary Fig. S2c). In conclusion, these data clearly indicated that upregulation of MCU enhances the mitochondrial Ca^2+^ uptake in CRC cells.

To investigate whether MCU overexpression results in mitochondrial Ca^2+^ overload, we further investigated the effect of MCU overexpression on mPTP opening using both mitochondrial swelling and calcein release assays (Supplementary Fig. S2d, e). Our data indicated that treatment with both MCU overexpression and cyclosporine A (CSA) had no effect on mitochondrial swelling and calcein release in CRC cells. Thus, suggesting that MCU-induced mitochondrial Ca^2+^ uptake and mitochondrial ROS production may be in the normal range and therefore, the functional status of mPTP is not affected.

### MCU-induced mitochondrial Ca^2+^ uptake promotes mitochondrial biogenesis in CRC cells

**Fig. 3:**
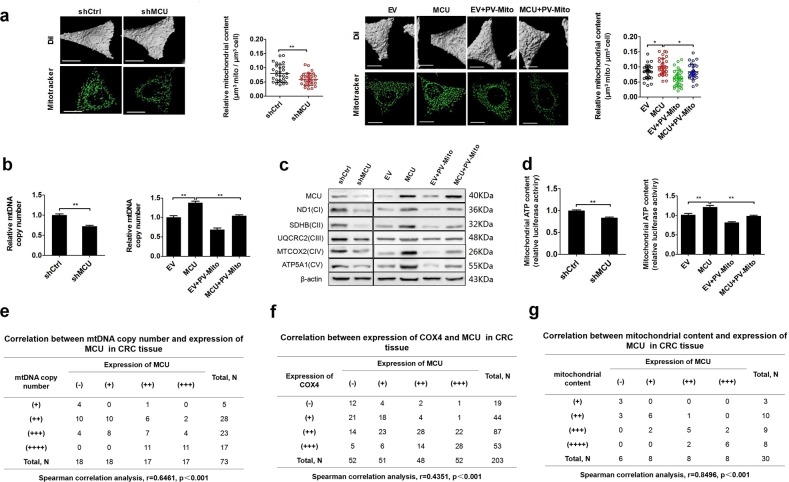
MCU-induced mitochondrial Ca^2+^ uptake promotes mitochondrial biogenesis in CRC cells. **a** Relative mitochondrial content (µm^3^ mitochondria per µm^3^ cell) was determined based on a mitochondrial three-dimensional model of confocal microscope images in LS174T cells, treated as indicated. MitoTracker (Green) was used to label mitochondria. **b** Relative mtDNA copy number was determined by RT-qPCR in LS174T cells, treated as indicated. **c** Western blotting analysis to measure the expression of oxidative phosphorylation related proteins in LS174 cells treated as indicated. **d** Mitochondrial ATP levels were determined using the ATP Determination Kit in LS174T cells, treated as indicated. **e** Correlation between relative mtDNA copy number and relative MCU expression in CRC tissue. The mtDNA copy number was divided into four levels based on the quartering of RT-qPCR results. As the quartiles are 3.21 (upper quartile), 4.12 (median) and 5.22 (lower quartile), RT-qPCR value <3.21, 3.21–4.12, 4.12–5.22, and >5.22 were defined as (+), (++), (+++), and (++++), respectively. **f** Correlation between relative cytochrome c oxidase subunit 4 expression and relative MCU expression in CRC tissue. **g** Correlation between relative mitochondrial content and relative MCU expression in CRC tissue. Mitochondrial content was divided into four groups based on the quartering of the relative mitochondrial content levels. As the quartiles are 0.15 (upper quartile), 0.21 (median) and 0.25 (lower quartile), relative mitochondrial content level <0.15, 0.15–0.21, 0.21–0.25, and >0.25 were defined as (+), (++), (+++), and(++++), respectively. *P < 0.05; **P < 0.01. MCU mitochondrial calcium uniporter, CRC colorectal cancer, mtDNA mitochondrial DNA, RT-qPCR reverse transcription-quantitative PCR, Ca^2+^ calcium

Several recent studies have reported the biological role of increased mitochondrial biogenesis in CRC tumorigenesis.^14,15^ Therefore, we further explored the correlation between the expression of MCU and mitochondrial biogenesis in CRC cells. As shown in Fig. 3a–d, LS174T MCU-knockdown cells exhibited a decrease in the relative mitochondrial content, relative mtDNA copy number, expression levels of oxidative phosphorylation (OXPHOS)-related proteins, and ATP production when compared with control cells. Similar results were obtained in LS174T cells treated with the [Ca^2+^]_m_ buffering using PV-Mito. In contrast, the opposite results were observed in MCU-overexpressing LS174T cells. Moreover, our data indicated that the [Ca^2+^]_m_ buffering by PV-Mito reversed the effects caused by MCU overexpression. IHC analysis also provided further supporting data, indicating that the protein expression level of MCU was positively correlated with protein expression level of cytochrome c oxidase subunit 4 (COX4), mtDNA copy number, and mitochondrial content in CRC tissues (Fig. 3e–g). All these findings suggest that MCU promotes mitochondrial biogenesis in CRC cells primarily by regulating the level of mitochondrial Ca^2+^.

### MCU-induced mitochondrial Ca^2+^ uptake promotes CRC growth in vitro

**Fig. 4:**
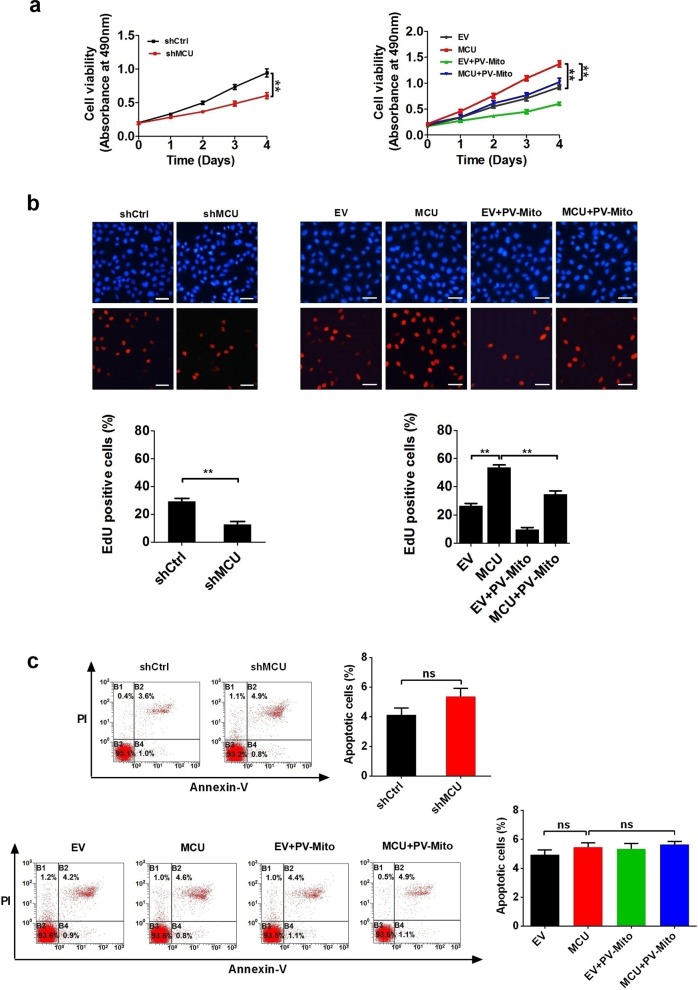
MCU-induced mitochondrial Ca^2+^ uptake promotes CRC growth in vitro. **a** MTS assays of LS174T cells, treated as indicated. **b** Representative images (upper panel) of EdU incorporation assays for cell proliferation in LS174T cells, treated as indicated and percentage of EDU-positive cells (lower panel), treated as indicated. **c** Flow cytometry analysis of cell apoptosis by Annexin V (an indicator of apoptosis) and PI staining in LS174T cells, treated as indicated. *P < 0.05; **P < 0.01. MCU mitochondrial calcium uniporter, CRC colorectal cancer, Ca^2+^ calcium

To determine the function of MCU-mediated mitochondrial Ca^2+^ uptake in CRC growth, we first established CRC cell lines with stable low or high expression of MCU by transfecting plasmids expressing MCU and its small-hairpin RNA (shRNA). As presented in Fig. 4a and Supplementary Fig. S3a, the cell viability assay showed that the decreased expression of MCU inhibited the growth of CRC cells compared with controls, whereas the opposite result was obtained when MCU expression was upregulated. Moreover, [Ca^2+^]_m_ buffering by PV-Mito significantly inhibited the growth of CRC cells and notably reversed the growth-promoting effect of MCU. Furthermore, the Edu incorporation assay indicated that MCU-knockdown cells had a lower percentage of proliferation compared with controls, whereas the opposite effects were observed in MCU-overexpressing cells (Fig. 4b and Supplementary Fig. S3b). Consistently, PV-Mito played a similar role in this assay. Moreover, flow cytometry analysis indicated that both MCU expression and [Ca^2+^]_m_ buffering by PV-Mito had no effect on CRC cell apoptosis (Fig. 4c). Taken together, these data indicated that MCU-mediated mitochondrial Ca^2+^ uptake facilitates CRC cell proliferation in vitro.

### MCU-induced mitochondrial Ca^2+^ uptake promotes CRC growth in vivo

**Fig. 5:**
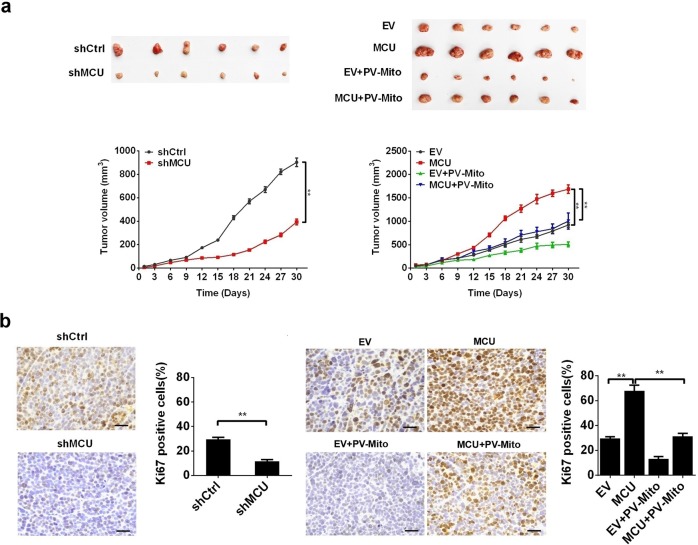
MCU-induced mitochondrial Ca^2+^ uptake promotes CRC growth in vivo. **a** Dissected tumors from sacrificed mice and tumor growth curves of subcutaneous xenograft tumor developed from LS174T cells, treated as indicated. **b** Representative immunohistochemistry staining images of Ki67 in xenograft tumors developed from LS174T cells, treated as indicated. *P < 0.05; **P < 0.01. MCU mitochondrial calcium uniporter, CRC colorectal cancer, Ca^2+^ calcium

The effect of MCU-mediated mitochondrial Ca^2+^ uptake on CRC cell growth was further studied in vivo by generating a CRC xenograft nude mice model. As shown in Fig. 5a, CRC xenografts with MCU knockdown showed a slower growth rate compared with the controls, whereas those overexpressing MCU exhibited a faster growth compared with the corresponding controls. PV-Mito treatment suppressed CRC growth and notably reversed the growth-promoting effect of MCU. Furthermore, IHC analysis (Fig. 5b) showed that the CRC xenografts with MCU knockdown or PV-Mito treatment exhibited a significantly lower percentage of Ki67-positive cells compared with the corresponding controls. In contrast, CRC xenografts overexpressing MCU had a significantly higher percentage of Ki67-positive cells compared with controls. These results support the conclusion that MCU-mediated mitochondrial Ca^2+^ uptake accelerates CRC growth in vivo by promoting CRC cell growth.

### Mitochondrial Ca^2+^ uptake promotes the dephosphorylation of TFAM to enhance the mitochondrial biogenesis

**Fig. 6:**
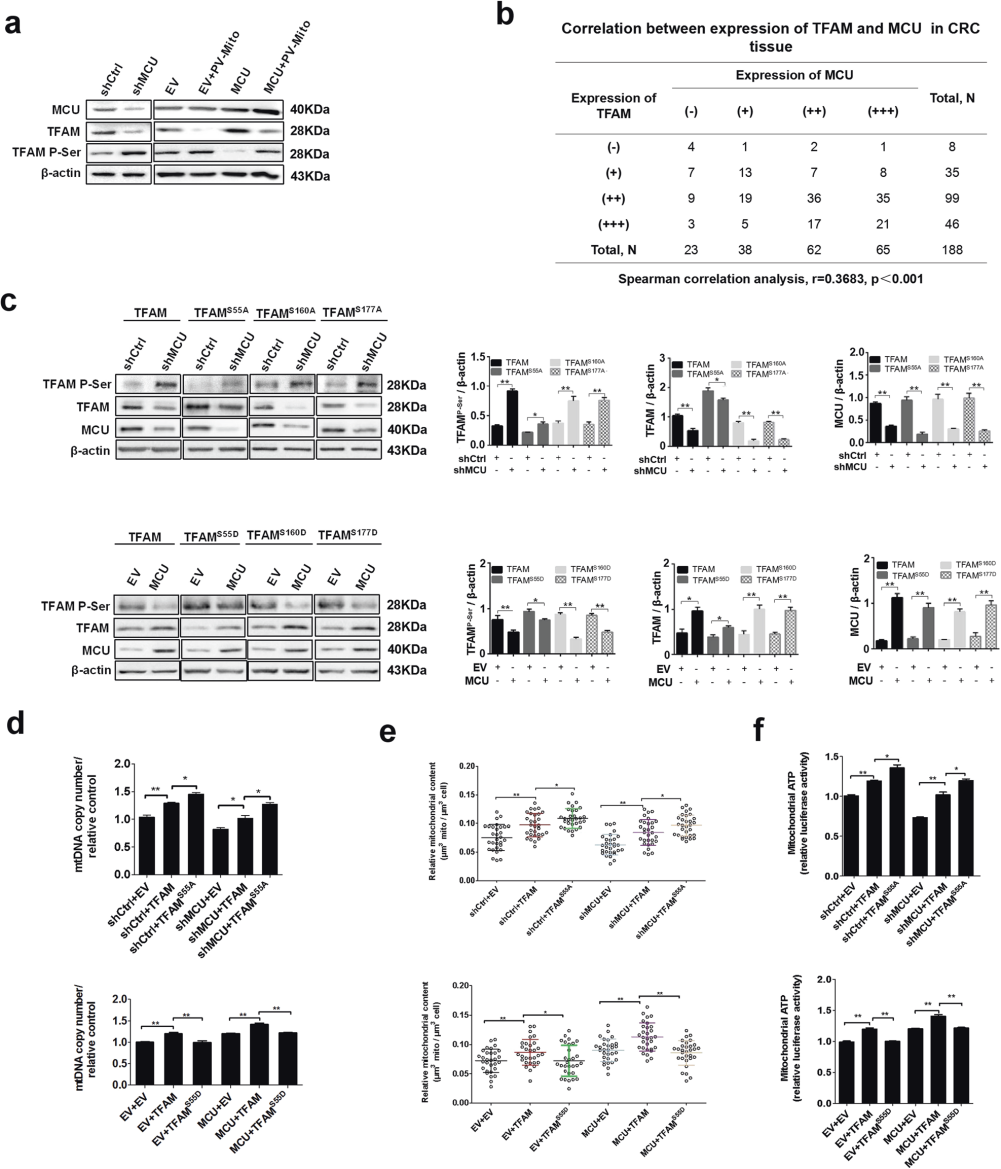
Mitochondrial Ca^2+^ promotes dephosphorylation of TFAM to enhance the mitochondrial biogenesis. **a** Western blotting analysis to measure protein expression levels of MCU, TFAM, and phosphorylated TFAM in LS174 cells, treated as indicated. **b** Correlation between relative TFAM expression and relative MCU expression in CRC tissue. **c** Western blotting analysis to measure protein expression levels of MCU, TFAM, and phosphorylated TFAM in LS174 cells, treated as indicated. TFAM S55A, mutation of serine-55 to alanine. Band intensity were quantified by densitometry and represented as the fold change of corresponding control value. **d** Reverse transcription-quantitative PCR analysis of relative mitochondrial DNA copy number in LS174T cells, treated as indicated. **e**, **f** Relative mitochondrial content and mitochondrial ATP levels were determined as mentioned above in LS174T cells treated as indicated. *P < 0.05; **P < 0.01. MCU mitochondrial calcium uniporter, CRC colorectal cancer, TFAM transcription factor A, mitochondrial, Ca^2+^, calcium

Previous studies have demonstrated that TFAM is a crucial regulator of mitochondrial biogenesis.^16–18^ Therefore, we attempted to examine whether TFAM is involved in the mitochondrial Ca^2+^-mediated mitochondrial biogenesis. The mRNA and protein expression levels of TFAM in MCU-knockdown or MCU-overexpressing CRC cells with MCU knockdown and overexpression were examined. We found that MCU expression had no effect on mRNA transcription of TFAM in CRC cells (Supplementary Fig. S4a). The expression of TFAM was downregulated in MCU-knockdown CRC cells compared with control cells, whereas the opposite result was observed in MCU-overexpressing CRC cells (Fig. 6a). IHC analysis also indicated that the protein expression level of MCU was positively correlated with the protein expression of TFAM in CRC tissues (Fig. 6b). Moreover, it has been reported that protein level of TFAM is regulated by the post-translational modification of phosphorylation, which leads to its degradation.^19^ Thus, we sought to determine whether mitochondrial Ca^2+^ exerts an effect on the phosphorylation of TFAM. Our data revealed that downregulation of MCU enhanced the phosphorylation of TFAM, whereas overexpression of MCU promoted dephosphorylation of TFAM in CRC cells (Fig. 6a). As expected, treatment with PV-Mito notably reversed the effect of MCU overexpression on TFAM expression and its phosphorylation (Fig. 6a). Furthermore, a site-directed mutagenesis assay of serine residues indicated that phosphorylation of TFAM was inhibited when serine-55 was mutated to alanine in MCU-knockdown CRC cells, whereas the phosphorylation state of TFAM was not affected when serine-160 or −170 was mutated to alanine (Fig. 6c), indicating that serine-55 of TFAM was the primary phosphorylation site. Additionally, a previous study has reported that mutation of serine-55 to aspartate can mimic the sustained phosphorylation of TFAM because the aspartate residue is negatively charged.^19^ Consistently, our western blotting analysis clearly demonstrated the phosphomimics of TFAM in MCU-overexpressing CRC cells when serine-55 was mutated to an aspartate residue. In contrast, phosphorylation of TFAM was not affected in MCU-overexpressing CRC cells when serine-160 or -177 were mutated to aspartate (Fig. 6c). Altogether, our data indicated that MCU-mediated mitochondrial Ca^2+^ uptake regulates the phosphorylation of TFAM primarily via serine-55, but not -160 and -177 (Fig. 6c). Collectively, our results suggested that mitochondrial Ca^2+^ may play a vital role in modulating the phosphorylation of TFAM.

Next, we examined whether MCU-mediated mitochondrial Ca^2+^ uptake promoted mitochondrial biogenesis by regulating the phosphorylation of TFAM. As shown in Fig. 6d–f and Supplementary Fig. S4b, c, TFAM overexpression resulted in a significant increase of the relative mtDNA copy number, relative mitochondrial content, expression levels of OXPHOS-related proteins and ATP production compared with the corresponding controls in MCU-knockdown or MCU-overexpressing CRC cells. This indicated that the protein expression of TFAM is essential for MCU-mediated mitochondrial biogenesis. Moreover, we found that the overexpression of TFAM^S55D^ resulted in a lower relative mtDNA copy number, relative mitochondrial content, expression levels of OXPHOS-related proteins and ATP production in MCU-knockdown or MCU-overexpressing CRC cells compared with the overexpression of wild type TFAM. In contrast, the overexpression of TFAM^S55A^ exhibited the opposite effect on mitochondrial biogenesis in MCU-knockdown or MCU-overexpressing CRC cells (Fig. 6d–f and Supplementary Fig. S4b, c). These findings supported the notion that TFAM phosphorylation plays a critical role in mitochondrial Ca^2+^-mediated mitochondrial biogenesis. In summary, our data revealed that mitochondrial Ca^2+^ may promote mitochondrial biogenesis primarily by regulating the phosphorylation of TFAM at serine-55 to affect the stability of TFAM in CRC cells.

Considering the close link between mitochondrial Ca^2+^ homeostasis and mitochondrial dynamics, we also investigated the effect of MCU-mediated mitochondrial Ca^2+^ uptake on the expression of proteins associated with mitochondrial dynamics. As shown in Supplementary Fig. S4d, CRC cells with MCU knockdown exhibited a decreased expression level of dynamin related protein 1 (Drp1) and phosphorylated Drp1 on Ser616 and an increased expression of OPA1 mitochondrial dynamin like GTPase. Similar results were obtained in CRC cells treated with PV-Mito. In contrast, the opposite results were observed in MCU-overexpressing CRC cells. Our data suggested that MCU-mediated mitochondrial Ca^2+^ uptake may promote mitochondrial fission and inhibit mitochondrial fusion, which is consistent with the findings of several previous reports.^20,21^

### Mitochondrial Ca^2+^-mediated mitochondrial biogenesis promotes CRC growth via ROS/NF-κB signaling

**Fig. 7:**
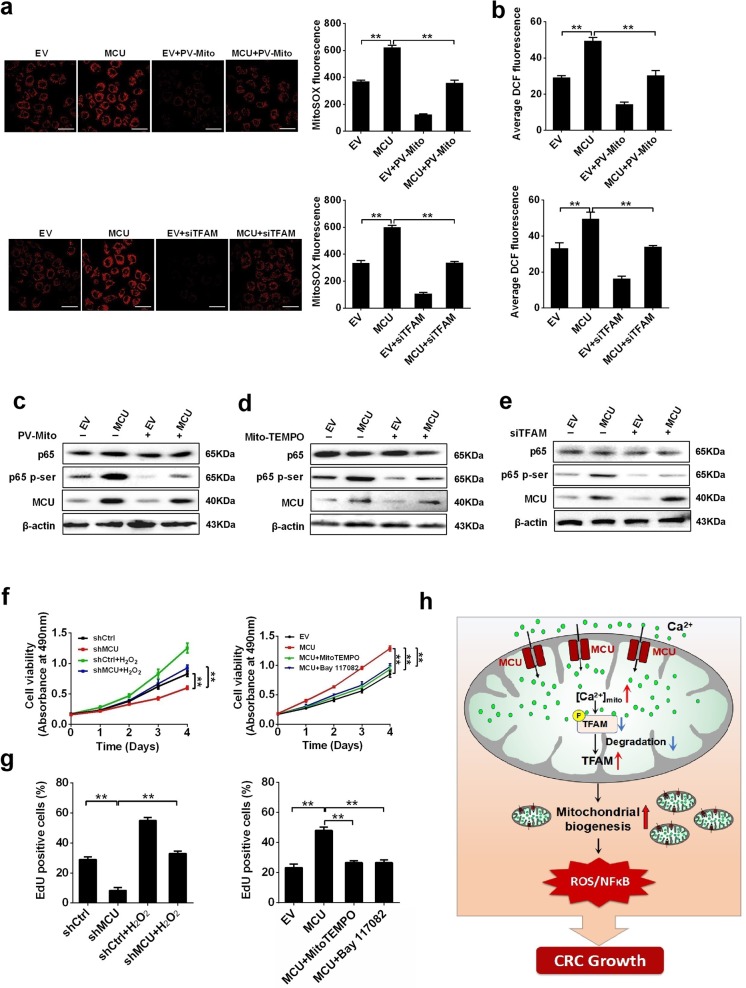
Mitochondrial Ca^2+^-mediated mitochondrial biogenesis promotes CRC proliferation by ROS/NF-kB signaling. **a** Immunofluorescence images (Left) of mitochondrial ROS (mROS) and MitoSOX fluorescence intensity (Right) in LS174T cells, treated as indicated. **b** Average DCF fluorescence intensity in LS174T cells treated as indicated. Western blotting analysis to measure the expression levels of p65 and pi-p65 in LS17T cells with MCU overexpression or treated with **c** PV-Mito, **d** Mito-TEMPO, or **e** siTFAM. **f** MTS assay to measure cell viability and **g** EdU incorporation assays to measure cell proliferation in LS174T cells, treated as indicated. **h** Schematic representation showing the underlying mechanism of MCU-mediated mitochondrial Ca2+ uptake in the promotion of CRC growth. *P < 0.05; **P < 0.01. MCU mitochondrial calcium uniporter, CRC colorectal cancer, TFAM transcription factor A, mitochondrial, ROS reactive oxygen species, PV-Mito expression vector encoding parvalbumin with mitochondria target sequence, si small interfering, Ca^2+^ calcium

Previous studies have demonstrated that [Ca^2+^]_m_ influences the production of ROS.^22^ Therefore, we explored whether MCU-mediated mitochondrial Ca^2+^ uptake would have an effect on ROS generation by TFAM-regulated mitochondrial biogenesis and thus promote CRC cell growth. As shown in Fig. 7a, b, when compared with controls, overexpression of MCU increased the mitochondrial and total ROS level in CRC cells, which was reversed by PV-Mito-mediated [Ca^2+^]_m_ buffering or TFAM knockdown. This finding indicated that mitochondrial biogenesis possesses a vital role in mitochondrial Ca^2+^-mediated generation of ROS. It has been well established that the ROS-activated NF-κB signaling pathway has a key role in various types of cancers.^23,24^ Thus, we aimed to examine whether the ROS/NF-κB pathway is involved in mitochondrial Ca^2+^-regulated CRC cell growth. Western blotting data implied that MCU overexpression increased the protein expression of phosphorylated p65, a key member of NF-κB signaling pathway, compared with the control (Fig. 7c–e). This effect of MCU overexpression on the activation of NF-κB signaling was reversed by PV-Mito mediated [Ca^2+^]_m_ buffering or mito-TEMPO, a mitochondrial ROS scavenger, or TFAM knockdown (Fig. 7c–e). Furthermore, treatment with H_2_O_2_ increased cell viability and the percentage of EDU-positive cells and clearly reversed the effect of MCU knockdown in CRC cells, whereas both the ROS scavenging by Mito-TEMPO and NF-κB-specific inhibitor Bay11–7082 decreased cell viability and the percentage of EDU-positive cells, clearly reversed the effect of MCU overexpression in CRC cells (Fig. 7f, g and Supplementary Fig. S5). Taken together, these data supported the notion that mitochondrial Ca^2+^-mediated mitochondrial biogenesis enhances CRC growth primarily via ROS/NF-κB signaling.

## Discussion

Previous studies indicated that mitochondrial Ca^2+^ entry regulated by the MCU complex is closely associated with cancer progression, with remarkably different underlying mechanisms depending on the type and stage of cancer. However, to date, the biological function of MCU in CRC remains unknown. In our study, we have obtained two major findings. First, we have demonstrated that upregulation of MCU promotes CRC cell growth via ROS/NF-κB signaling. More importantly, to the best of our knowledge, we have for the first time established a link between the upregulation of MCU and mitochondrial biogenesis by mitochondrial Ca^2+^-mediated dephosphorylation of TFAM, which provides a novel insight into the regulation of mitochondrial functions.

A number of studies have demonstrated that deregulation of MCU is associated with different types of cancer.^7^ For instance, it has been reported that the expression of MCU is elevated in various types of cancer, including breast cancer^25^ and hepatocellular carcinoma.^11^ Consistently, our data indicated that the mRNA and protein expression levels of MCU were frequently upregulated in CRC cells and tissues, which then contributed to poorer OS of patients with CRC. We further confirmed our results in the Human Protein Atlas database,^26^ indicating that MCU is overexpressed at both the mRNA and protein level in CRC. However, Marchi et al.^27^ reported an inconsistent result, indicating that protein expression of MCU is decreased in CRC. This discrepancy may be caused by selection bias of samples such as different stages of CRC and treatment procedures or use of a different antibody for IHC, which needs to be clarified in future studies. A recent study has demonstrated that alternations in the stoichiometry of MCU complexes causes a change in the Ca^2+^ influx into the mitochondria.^28^ Furthermore, Julia et al. reported that the MICU1 functions as a gatekeeper to inhibit mitochondrial Ca^2+^ overload.^29^ Our data indicated that the mRNA expression of MICU1, but not other members of the MCU complex is downregulated in CRC tissues, suggesting that MICU1 may function together with MCU to regulate the level of mitochondrial Ca^2+^ in CRC cells.

Previous studies indicated that the MCU complex possesses distinct functions in different types of cancer. For instance, Tosatto et al.^12^ reported that silencing MCU in breast cancer cells results in decreased ROS production and expression of hypoxia-inducible factor-1α (HIF-1α), which then leads to reduced tumor growth. A recent study also revealed that downregulation of MCU suppressed cell motility and reduced tumor growth in triple-negative breast cancer by regulating store-operated Ca^2+^ entry.^30^ Moreover, a study from our group revealed that MCU promotes the metastasis of HCC cells via the nicotinamide adenine dinucleotide + /sirtuin 3/superoxide dismutase 2 signaling pathway.^11^ To the best of our knowledge, the present study is the first to provide evidence that MCU-mediated mitochondrial Ca^2+^ uptake is crucial for CRC cell growth by increasing mitochondrial biogenesis and ROS/NF-κB signaling. Previous studies have indicated that mitochondrial Ca^2+^ overload is closely involved in ROS production and the decrease of mitochondrial membrane potential, thus leading to cell death.^31^ In comparison, accumulating evidence indicates that mitochondrial Ca^2+^ uptake mediated by upregulated MCU significantly promotes cell proliferation in cancers. Li et al^32^. showed that MCU overexpression is associated with the glioblastoma cell proliferation but not the induction of cell death. Curry et al.^9^ demonstrated that MCU overexpression is a feature of breast cancers and offers a survival advantage against cell death pathways. Zeng et al.^33^ reported that RIPK1 interacts with MCU to promote colorectal cancer cell proliferation by increasing mitochondrial Ca^2+^ uptake and energy metabolism. Consistent with these reports, our data indicated that treatment with MCU overexpression had no effect on mitochondrial swelling and calcein release in CRC cells, suggesting that MCU-induced mitochondrial Ca^2+^ uptake and mitochondrial ROS production may be in a normal range, and thus the functional status of mPTP is not affected. Our data have also provided further support in showing that MCU overexpression had no effect on the apoptosis of CRC cells. These findings further suggested that the functional role of MCU-mediated mitochondrial Ca^2+^ uptake may be context dependent or cancer cell type specific.

Our data showed that MCU-mediated mitochondrial Ca^2+^ uptake notably enhanced mitochondrial biogenesis by regulating the dephosphorylation of TFAM and thus increasing its stability. Furthermore, it has been recently reported that the stability of TFAM in mitochondria is mediated by post-translational phosphorylation of serine-55.^19^ Consistently, our site-directed mutagenesis analysis indicated that TFAM was phosphorylated at serine-55. Furthermore, our data revealed that phosphorylation of TFAM led to its degradation, while dephosphorylation of TFAM via the mutation of serine-55 to alanine greatly enhanced TFAM stability, which is consistent with previous studies.^19,34^ It has been reported^34^ that phosphodiesterase 2A is involved in the regulation of the protein kinase A activity, which is responsible for the phosphorylation of TFAM in a number of cases.^19^ Therefore, we hypothesized that both enzymes may be related to the dephosphorylation of TFAM induced by mitochondrial Ca^2+^, which warrants further investigation in the future. Our data further demonstrates that mitochondrial Ca^2+^ uptake increases mitochondrial biogenesis by promoting dephosphorylation of TFAM. This is consistent with previous findings, indicating that TFAM is an essential regulator of mitochondrial biogenesis.^18^ Our results provide further understanding of the molecular mechanism underlying mitochondrial Ca^2+^-mediated mitochondrial biogenesis. Several studies have reported the increase of mitochondrial biogenesis in CRC cells. For example, Witherspoon et al.^14^ indicated that the constitutive expression of ETHE1 increases aerobic glycolysis (“Warburg effect”), oxidative phosphorylation, and mitochondrial biogenesis in colorectal cancer (CRC) cell lines.^35^ Yang and colleagues^36^ suggested that mitochondrial biogenesis and maintenance may play an important part in tumor cell survival during CRC progression. In our study, our data showed that upregulated MCU promotes mitochondrial biogenesis in CRC cells. Very similarly, Zeng et al.^33^ also reported that basal and maximal respirations are significantly higher in MCU-overexpressing HT-29 cells than that in controls.

It is generally well accepted that dividing cells, including cancer cells, meet their energy demands by reprogramming their cell metabolism such as altering mitochondrial dynamics.^37,38^ Our data suggested that MCU-mediated mitochondrial Ca^2+^ uptake may promote mitochondrial fission and inhibit mitochondrial fusion, which is in agreement with several previous reports.^20,21^ Over the past decades, multiple studies have reported that mitochondrial biogenesis and quality control are often upregulated in various types of cancer, including breast cancer, lung cancer, and hepatocellular carcinoma.^39–42^ However, the mechanism underlying enhanced mitochondrial biogenesis in CRC remains obscure. In the present study, we showed that MCU-mediated Ca^2+^ uptake played an essential role in mitochondrial biogenesis via dephosphorylation of TFAM. To the best of our knowledge, our work provides the first mechanistic insights into mitochondrial Ca^2+^-mediated mitochondrial biogenesis in CRC.

Growing evidence suggests that accumulation of Ca^2+^ in mitochondria leads to ROS production.^43,44^ In the present study, we showed that the forced expression of MCU in CRC cells greatly enhanced mitochondrial Ca^2+^ uptake and a markedly increased the production of ROS. Moreover, growing evidence has demonstrated that there is an association between ROS and NF-κB signaling.^45^ For instance, Takada et al.^46^ demonstrated that ROS influences the activation of the NF-κB pathway primarily by inhibiting the phosphorylation of nuclear factor of kappa light polypeptide gene enhancer in B-cells inhibitor, alpha,^46^ and that ROS also results in S-glutathionylation of IκB kinase β (IKKβ) on cysteine-179 and then suppresses IKKβ activity.^47^ Consistently, our data revealed that increased mitochondrial biogenesis promoted ROS production to activate NF-κB signaling, which in turn facilitated CRC growth in vitro and in vivo.

Several studies have reported that Ru360 is a highly potent and selective MCU inhibitor.^48^ In our previous publication,^11^ blocking of MCU activity by Ru360 has been verified in HCC cells. Moreover, our data reported that MCU-mediated mitochondrial Ca^2+^ uptake is effectively blocked by Ru360 in CRC cells. These results suggested a potential application as an antineoplastic compound. However, other studies have also demonstrated that its general applicability is limited by several shortcomings.^49^ For example, this compound is impermeant to the plasma membrane in specific cell lines. Furthermore, its synthesis is challenging and low-yielding. Fortunately, recently the structural data of MCU has become available,^50^ which is beneficial to elucidate the structural basis of Ru360 blocking and could be used to improve its potency and selectivity by developing its structural analogs.

In conclusion, we systematically investigated the functional role of MCU-mediated mitochondrial Ca^2+^ homeostasis in promoting CRC cell growth in vitro and in vivo. We demonstrated that MCU-mediated mitochondrial Ca^2+^ uptake significantly promotes mitochondrial biogenesis by promoting the dephosphorylation of TFAM. Furthermore, we also demonstrated that increased mitochondrial Ca^2+^ uptake promotes CRC growth by activating NF-κB signaling via ROS. Our findings identify a novel mechanism underlying MCU-mediated mitochondrial Ca^2+^ uptake in facilitating CRC cell growth.

## Materials and methods

### Cell culture and tissue collection

Human CRC cell lines COLO205, LS174T, LoVo, HCT-8, Caco-2, SW620, DLD-1, and HT-29 and were purchased from The American Type Culture Collection (USA) and routinely cultured at 37 ˚C and 5% CO_2_ in Dulbecco’s modified Eagle’s medium (DMEM) or RPMI-1640 medium, which was supplemented with 10% fetal bovine serum. Tumor tissues and paired adjacent non-tumor tissues were collected from 203 patients who had undergone surgery and had no treatment before collection of tissue samples at Tangdu Hospital affiliated with Fourth Military Medical University. Supplementary Table S1 lists the distribution of clinical characteristics of 203 patients with CRC. This study was performed with approval from the Ethics Committee of Fourth Military Medical University (Permission number: KY20173189–1; Date issued: 2017-03-06).

### Knockdown and overexpression of target genes

To generate shRNA expression vectors, a shRNA targeting the human MCU mRNA sequence and a control shRNA were cloned into the pSilencer™ 3.1-H1 puro vector (Ambion; Thermo Fisher Scientific, Inc., USA). The shRNA sequences are listed in Supplementary Table S2. To overexpress the target genes, complementary DNA (cDNA) derived from LS174T cells was used as a DNA template to amplify MCU and TFAM using the primers listed in Supplementary Table S2. The target genes were then cloned into a pcDNA3.1(+) vector (Invitrogen; Thermo Fisher Scientific, Inc., USA). The expression plasmid of PV-Mito encoding mitochondrial Ca^2+-^binding protein was provided by Dr. Atsushi Miyawaki (RIKEN Brain Science Institute, Japan). Cells were seeded in 6-well plates until they reached 60% to 80% confluence for transfection of vectors or small-interfering RNAs (siRNAs) with Lipofectamine^®^ 2000 reagent (Thermo Fisher Scientific, Inc., USA) according to the manufacturer’s protocols. All siRNAs were synthesized by Shanghai GenePharma Co., Ltd., (China) and their sequences are listed in Supplementary Table S2.

### Measurement of mtDNA content by RT-qPCR

E.Z.N.A Tissue DNA Kit (Omega Bio-Tek, Inc., USA) was used to extract genomic DNA, according to the manufacturer’s instructions. An RT-qPCR-based method was employed to measure the relative mtDNA copy number, as previously described.^37^

### Quantitative reverse transcription PCR (RT-qPCR) analyses

Total RNA was isolated from cultured CRC cells or human CRC tissues and reversely transcribed. RT-qPCR was performed as previously described.^21^ Relative mRNA expression levels were quantified by the 2^−ΔΔCt^ method. Experiments were performed in triplicate and GAPDH was used as an internal control. Supplementary Table S2 lists the primer sequences used for RT-qPCR analysis.

### Western blotting analysis

Western blotting assays were performed based on regular procedures. Briefly, total proteins from lysed cell samples were analyzed on SDS-polyacrylamide gel electrophoresis (SDS-PAGE) and electrophoretically separated proteins were subsequently transferred to a polyvinylidene fluoride (PVDF) membrane. The membrane with transferred proteins was then probed with a specific primary antibody overnight at 4 ˚C and incubated with a horseradish peroxidase-conjugated-secondary antibody for 2 h at room temperature. β-actin was used as a loading control in western blotting analysis. The membrane was then visualized using the enhanced chemiluminescence system (Pierce; Thermo Fisher Scientific, Inc., USA). The antibody dilutions used are listed in Supplementary Table S3.

### Immunohistochemical (IHC) staining

IHC analysis and quantification of IHC staining score was evaluated as previously described.^24^ The expression of proteins was determined by two independent pathologists who were blinded to the clinical characteristics of the patients.

### Cell viability and apoptosis assays

Cell viability and apoptosis experiments were carried out as previously described.^24^

### Cell proliferation assay

Cell-Light^TM^ EdU DNA Cell Proliferation Kit (Guangzhou Ribobio Co., Ltd, China) was used to detect cell proliferation. As previously stated,^51^ EdU reagent was employed to treat cells 48 h after transfection, followed by treatment with a permeabilization and phosphate-buffered saline (PBS) buffer. Cells were then visualized under a fluorescence microscope after staining with Apollo reagent for 30 min.

### Site-directed mutagenesis

Site-directed mutagenesis was carried out using the mutagenesis kit (Beyotime Institute of Biotechnology, China) according to the manufacturer’s instructions. In brief, the PCR primers (Supplementary Table S2) containing desired mutation sites were used to establish of plasmid constructs expressing mutant TFAM, which were referred to as pcDNA-TFAM^S55D^ and pcDNA-TFAM^S55A^. The plasmid pcDNA3.1-TFAM was used as the DNA template for PCR amplification, according to the reaction conditions recommended by the mutagenesis kit. The resulting constructs, pcDNA-TFAM^S55D^ and pcDNA-TFAM^S55A^, were confirmed by DNA sequencing (Shanghai Shenggong, Biology Engineering Technology Service, Ltd., China). Proteins resulting from these plasmids are referred to as TFAM^S55A^ and TFAM^S55D^.

### Measurement of mitochondrial Ca^2+^

The amount of Ca^2+^ in mitochondria was determined as described previously.^24^ In brief, cells were transfected with a plasmid carrying mitochondrial matrix-targeted fluorescent tagged inverse pericam, which was referred to as mitopericam. A confocal laser scanning microscope FV1000 (Olympus Corporation, Japan) was then employed to detect the cells. To measure dynamic mitochondrial Ca^2+^, histamine (10 µM) was added after 30 s of baseline recording, 380 nm and 490 nm excitation filters were utilized in combination with 540 nm emission filter and photos were recorded every 3 s.

### Detection of mitochondrial swelling

Freshly isolated mitochondria were added to buffer containing 120 mM KCl, 10 mM Tris, 20 mM MOPS, and 5 mM KH_2_PO_4_ (pH 7.4) to achieve a final concentration of 0.25 mg/mL mitochondria. Mitochondrial swelling was detected by monitoring the decrease in light scattering. The absorbance at 530 nm was monitored every 30 s for 10 min. In addition, the relative absorbance was also compared for samples at 0 min and 60 min, respectively. In order to determine PTP-dependent mitochondrial swelling, CSA (200 nM), which is a cis-trans isomerase activity inhibitor of cyclophilin D (CypD), was used to inhibit PTP opening, while FCCP (5 µM), which causes depolarization of mitochondrial inner membrane, was used to promote PTP opening.

### Calcein release assay

Cells were suspended in complete medium at a final concentration of 1 × 10^6^/mL, and then transferred to a 96-well plate (100 µL/well) for 4 h incubation with 5 µM fluorescent dye calcein-AM (Thermo Fisher Scientific, Inc., USA) in a CO_2_ incubator at 37 ˚C. Cells were then washed three times (5 min each time) by PBS in presence of CoCl_2_ (1 mM) to quench the cytosolic compartments. Finally, cells were re-suspended in 100 μL PBS for data collection. A microplate reader (Bio-Rad Laboratories, Inc., USA) was employed to measure calcein fluorescence every 5 min over 25 min. In addition, the relative calcein fluorescence was measured at 0 and 60 min, respectively.

### Measurement of mitochondrial mass

A three-dimensional (3D) model of mitochondria was constructed using the method described by Cheverollier et al.^52^ Briefly, 100 nM Mitotracker^TM^ Green was used to label mitochondria, and DMEM without red phenol was employed to wash the cells. For imaging, coverslips were mounted in an incubation chamber placed on the stage of an inverted microscope. An inverted wide-field Leica microscope equipped with a high-sensitivity CCD camera was employed to obtain images. An average of 30 image planes were obtained along the *z-*axis at 0.2 µm increments. Three-dimensional data processing and morphometric analysis was performed using the Imaris 7.1.1 software (Bitplane, USA).

### Mitochondrial content analyses

Mitochondrial content was determined using the method described previously.^24^ Briefly, glutaraldehyde was used to fix human CRC tissues. The specimens were then postfixed with osmium tetroxide, dehydrated with alcohol and embedded in araldite. Uranyl acetate and lead citrate were used to stain the thin sections, followed by visualization under a Tecnai G2 electron microscope (FEI; Thermo Fisher Scientific, Inc., USA). The area of mitochondria and CRC cells were determined using FIJI software (NIH, USA). Transmission electron microscopy images were examined by two independent pathologists, who were blinded to clinical data. The mitochondrial content was calculated by the ratio of mitochondrial area to CRC cell area.

### Detection of reactive oxygen species

Cellular ROS and mitochondrial reactive species (mitoROS) were detected using the fluorescence probe DCFH-DA (Beyotime Institute of Biotechnology, China) and mitoSOX fluorescence probe (Invitrogen; Thermo Fisher Scientific, Inc., USA), respectively, following the protocols described previously.^10^ ImagePro image analysis software (Media Cybernetics, Inc., USA) was used to capture and process the images.

### In vivo subcutaneous xenograft models

The dorsal right flank of five-week-old Balb/c nude mice (six per group) was subcutaneously injected with LS174T cells with low or high expression levels of MCU. The tumor volume was measured every three days for four weeks. Then mice were sacrificed to measure the wet weight of the excised tumors. All animal experiments were conducted according to the guidelines of the Institutional Animal Care and Use Committee of the Fourth Military Medical University (Permission number: IACUC-20170105; Date issued: 2017-01-01).

### Immunoprecipitation assay

The phosphorylation of TFAM was assayed using the immunoprecipitation assay. Cells were lysed and incubated with 200 μL Protein A beads (Santa Cruz Biotechnology) supplemented with 50 μg anti-TFAM antibody overnight. Normalized amounts of total lysates or immunoprecipitated samples were analyzed by SDS-PAGE and western blotting with phosphorylated antibody.^19^

### Statistical analysis

All statistical analyses were performed using SPSS 17.0 software (SPSS, Inc., USA). Data are shown as the mean ± SD from three independent experiments, where appropriate. Student’s *t*-test was employed to analyze the significant differences between two groups. Paired two-tailed *t*-tests were employed to compare the differences between tumor tissue and adjacent non-tumor mucosa. The correlations between measured variables were analyzed by Spearman’s correlation. *P* > 0.05 was considered a statistically significant difference. For prognosis analysis, MCU expression was first categorized into high or low level by the median value of the IHC score. The OS and RFS in patients with CRC who had high or low MCU expression were compared using Kaplan–Meier survival curves and log-rank test.

## Supplementary information

Supplementary Information
