# Supplementary material for: MCU-induced mitochondrial calcium uptake promotes mitochondrial biogenesis and colorectal cancer growth
Source: Signal Transduct Target Ther. 2020 May 5;5:59. doi: 10.1038/s41392-020-0155-5 (PMC7200750; doi:10.1038/s41392-020-0155-5)
Supplement: Supplementary file 1 — Supplementary Information [file 41392_2020_155_MOESM1_ESM.doc]

Supplementary Materials for

**MCU-induced mitochondrial calcium uptake promotes mitochondrial biogenesis and colorectal cancer growth**

Yang Liu^1,2#^, Mingpeng Jin^1#^, Yaya Wang^1,3#^, Jianjun Zhu^4^, Rui Tan^5^, Jing Zhao^1^ Xiaoying Ji^1^, Chao Jin^6^, Yongfeng Jia^2^, Tingting Ren^6^*, Jinliang Xing^1^*

Correspondence to: Jinliang [Xing (xingjl@fmmu.edu.cn)](mailto:Xing(xingjl@fmmu.edu.cn)) or Tingting Ren (rtt419@fmmu.edu.cn)

**This PDF file includes:**

Figures. S1 to S5

Tables S1 to S3


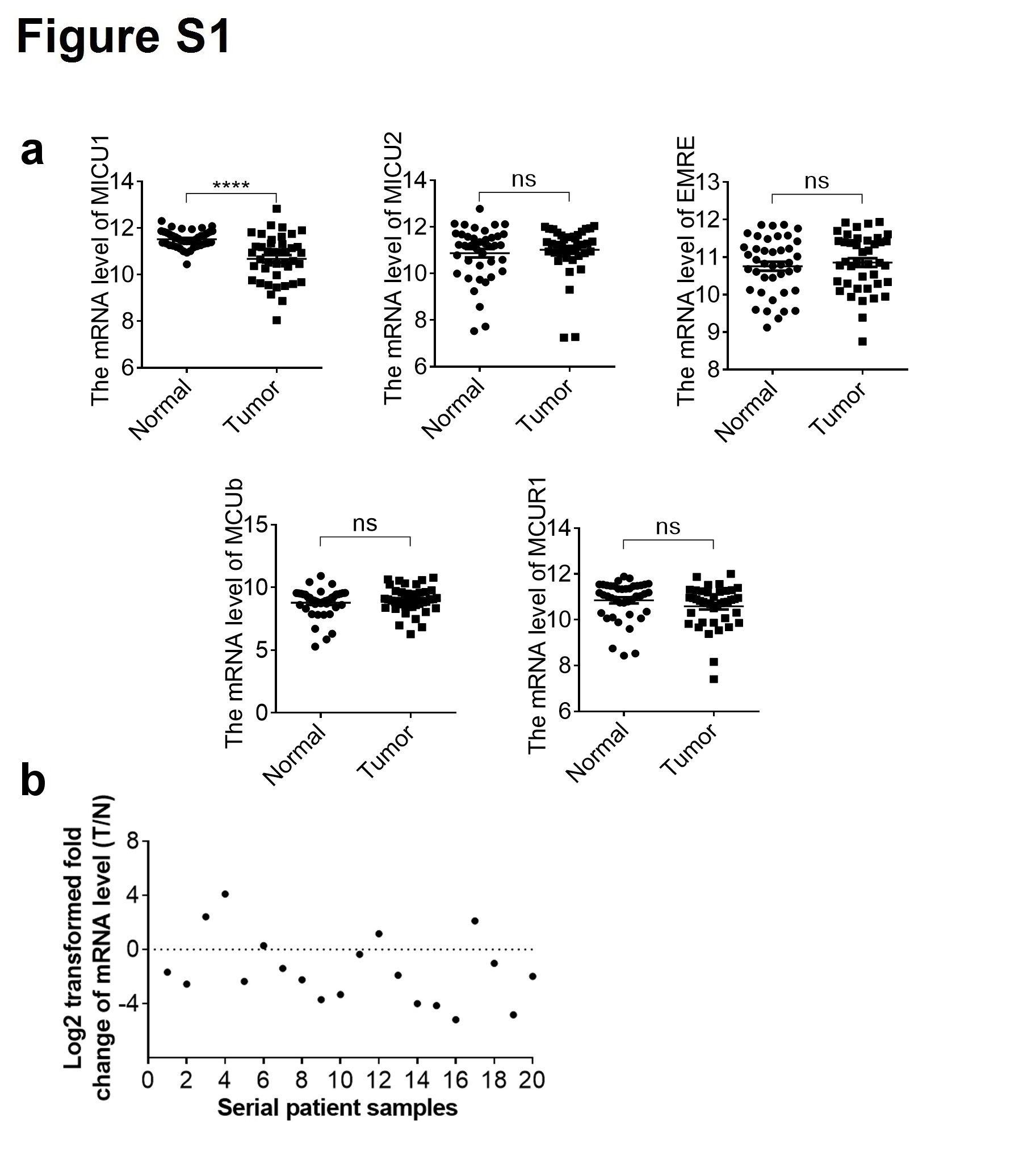


**Figure S1. (a)** Relative mRNA expression levels of MICU1, MICU2, EMRE, MCUb and MCUR1 analyzed using the RNA-seq data from TCGA database. **(b)** Quantitative reverse transcription PCR analysis for relative mRNA expression level of MICU1 in 20 paired tissues. The ratio of relative mRNA expression between tumor and normal tissues was log2-transformed (T, tumor; N, normal). **** *P*<0.0001; ns: *P*＞0.05.


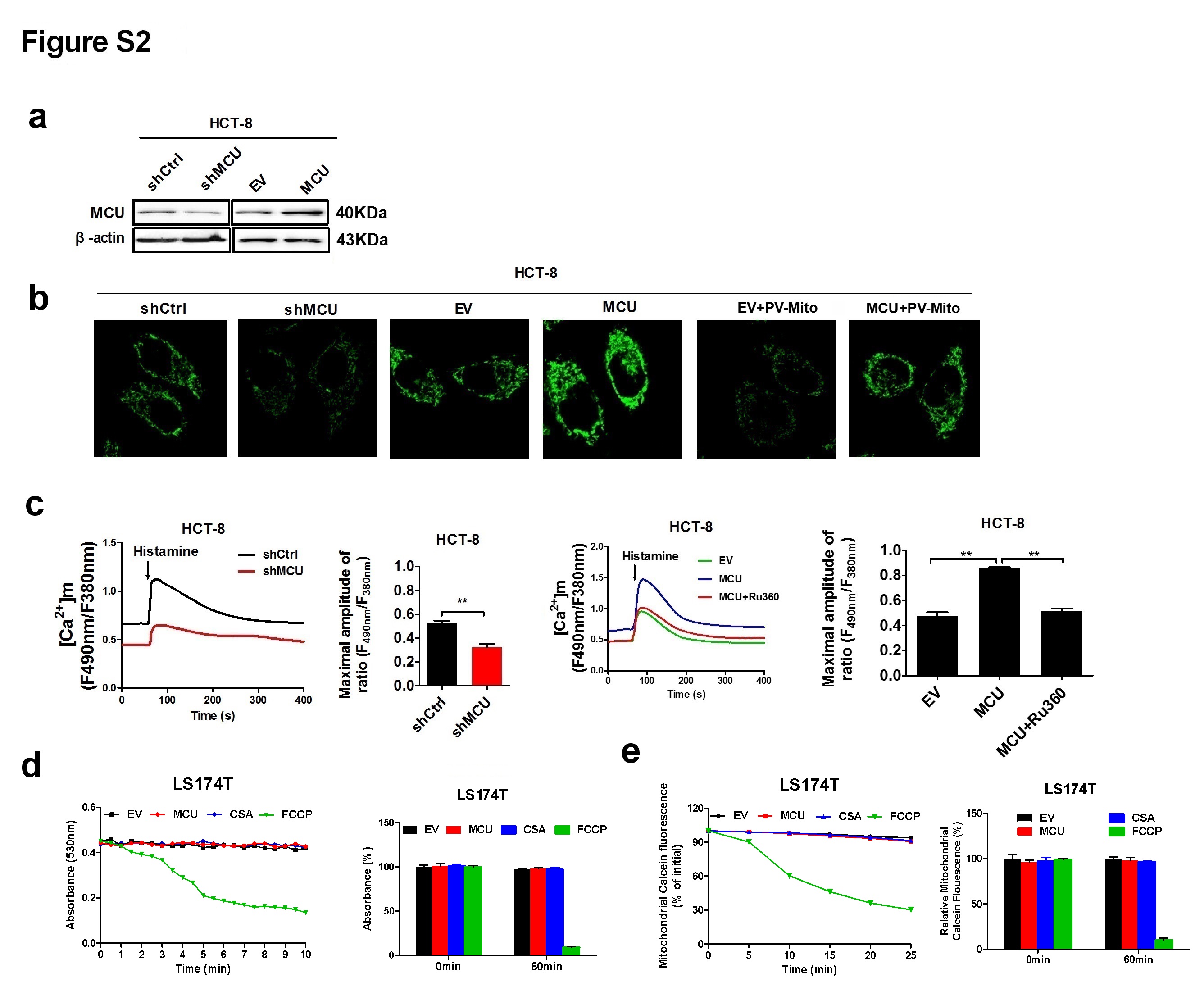


**Figure S2. (a)** Western blotting analysis for MCU protein expression in HCT-8 cells with treatments as indicated (shCtrl, control shRNA; shMCU, shRNA against MCU; MCU, expression vector encoding MCU; EV, empty vector). **(b)** Confocal microscope images of mitochondrial Ca^2+^ levels ([Ca^2+^]m) using MitoPericam (Green) to label mitochondria in HCT-8 cells treated as indicated. **(c)** Confocal microscope analysis of [Ca^2+^]m in CRC cells treated as indicated. Ruthenium 360 (Ru360, 10µM) was used to inhibit MCU activity. PV protein with mitochondrial translocation signal was used to buffer mitochondrial Ca^2+^. **(d)**Representative curves (Left) of PTP opening determined by monitoring the Ca^2+^-induced decrease of light scattering at 530 nm over 10 min in LS174T cells treated with EV (control group), MCU (MCU overexpression group), CSA (PTP inhibitor, 200nM) and FCCP (Mitochondrial oxidative phosphorylation inhibitor, 5µM) and quantitative data (Right) shown as a ratio of optical density (OD), which is defined as A530 of any other samples at any time points/mean A530 of EV group at 0 min. **(e)** Curves of mitochondrial calcein fluorescence (Left) measured over 25 minutes in LS174T cells treated as indicated. Relative calcein fluorescence (Right) was measured for samples at 0 min and 60 min and data were represented as the value of any other samples at 0 min and 60 min/mean value of EV group at 0 min ×%. in the same way as (d). * P<0.05; ** P<0.01


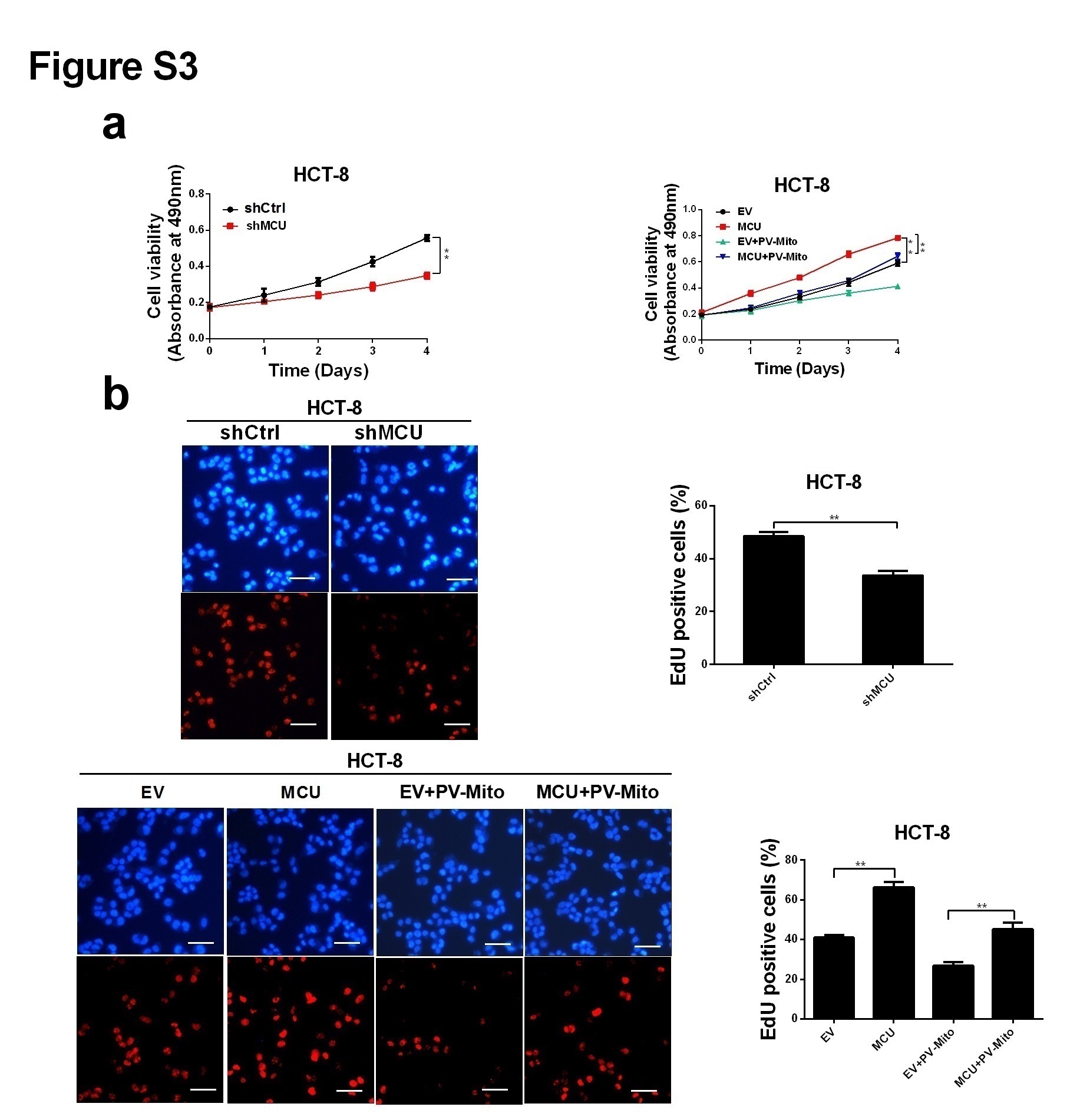


**Figure S3**. MTS assay for cell viability in HCT-8 cells treated as indicated. **(a)** Representative images of EdU incorporation assays for cell proliferation in HCT-8 cells with treatments as indicated. **(b)** Representative images (Left) of EdU incorporation assays for cell proliferation and percentage of EDU-positive cells (Right) in HCT-8 cells treated as indicated. * *P<*0.05*;* ** *P<*0.01


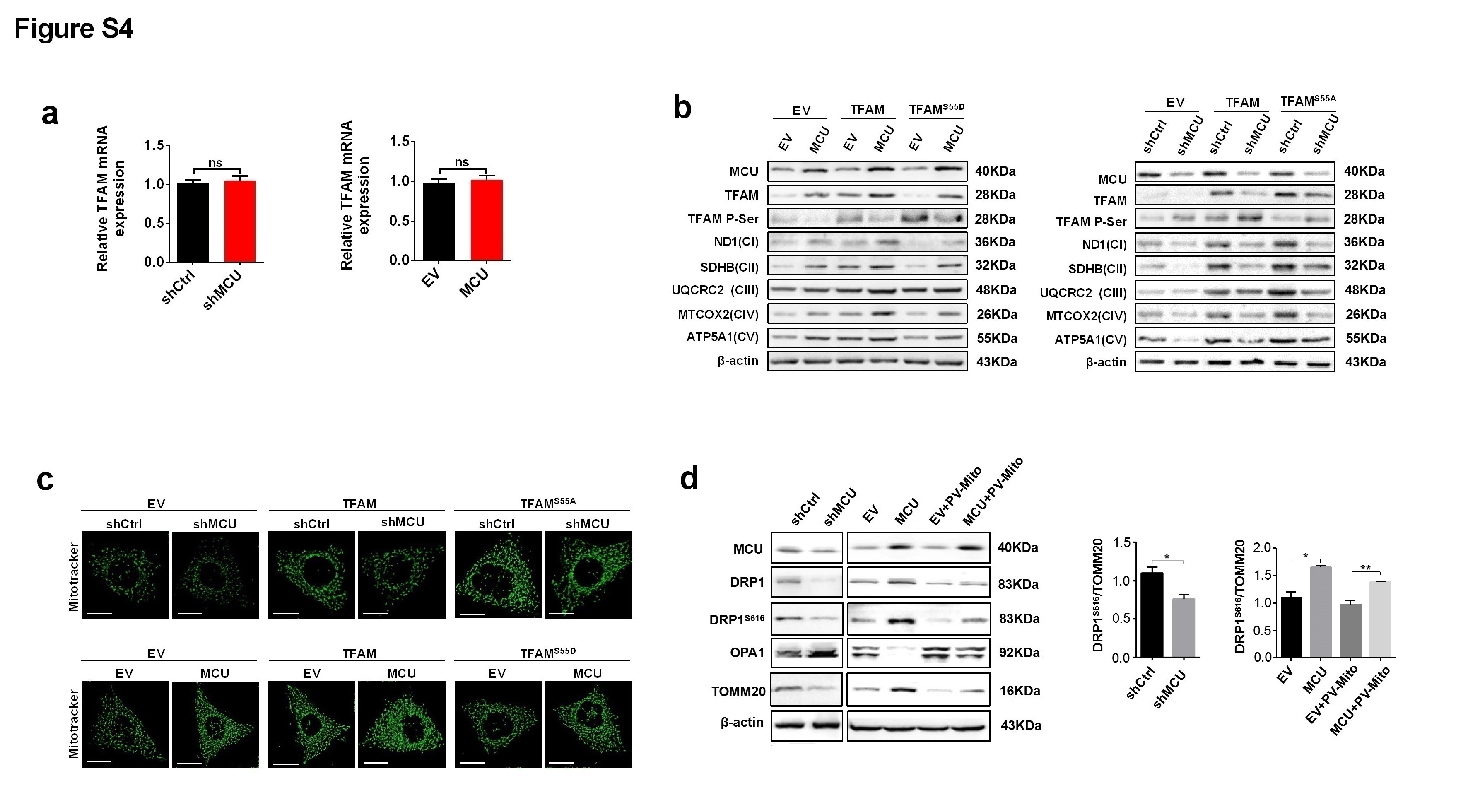


**Figure S4. (a)** RT-qPCR analysis for mRNA expression level of TFAM in LS174T cells treated as indicated. **(b)** Western blotting analysis for expressions of oxidative phosphorylation proteins including ND1, SDHB, UQCRC2, MTCOX2 and ATP5A1 in LS174T cells with treatments as indicated (TFAM, expression vector encoding TFAM; TFAM^S55A^, serine to alanine mutation; TFAM^S55D^, serine to aspartate mutation). **(c)** Confocal microscope images of mitochondria in LS174T cells treated as indicated. **(d)** Western blotting analysis for expressions of proteins including DRP1, DRP1^S616^, OPA1, TOMM20 and β-actin in LS174T cells treated as indicated.


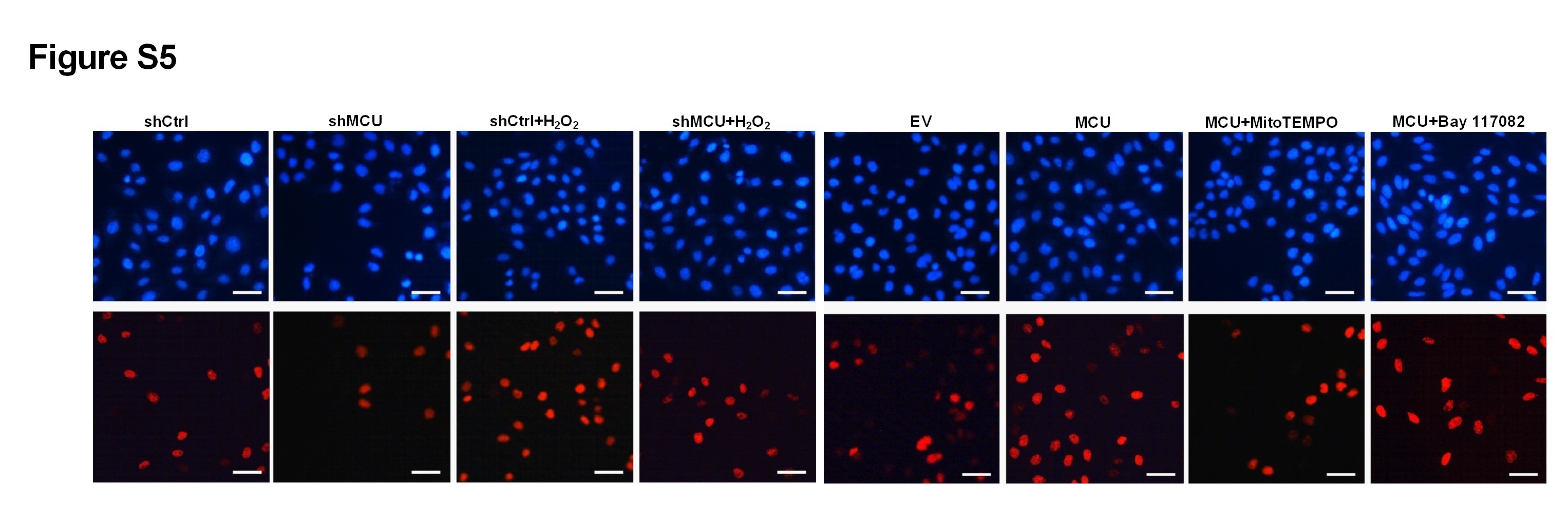


**Figure S5.** Representative images of EdU incorporation assays for cell proliferation in LS174T cells with treatments as indicated. DAPI was used to stain nuclei (Blue).

**Supplemental table 1. Distribution of CRC patients’ characteristics.**

| **Parameter** | **All patients n (%)**  **n=203** |
| --- | --- |
| **Gender, n (%)** |  |
| Female | 91(44.8%) |
| male | 112(55.2%) |
| **Age, Median(Range）** | 61(28-90) |
| **Locus, n (%)** |  |
| Colon | 96(47.3%) |
| Rectum | 107(52.7%) |
| **TNM stage, n (%)** |  |
| Ⅰ+Ⅱ | 133(65.3%) |
| Ⅲ+Ⅳ | 70(34.7%) |

**Supplemental table 2. Sequences of primers.**

| **1.Primers used in qPCR analysis** | | |
| --- | --- | --- |
| **Gene** | **Primer pair** | **Primer sequence** |
| MCU | Forward | TCCAGAAGCCAGAGACAGAC |
|  | Reverse | TGTCGGAGAGGCAGATGTAC |
| GAPDH | Forward | GGAGCGAGATCCCTCCAAAAT |
|  | Reverse | GGCTGTTGTCATACTTCTCATGG |
| ND1 | Forward | CCCTAAAACCCGCCACATCT |
|  | Reverse | GAGCGATGGTGAGAGCTAAGGT |
| HGB | Forward | GCTTCT GACACAACTGTGTTCACTAGC |
|  | Reverse | CACCAACTTCATCCACGTTCACC |
| TFAM | Forward | CCATCTACCGACCGGATGTTA |
|  | Reverse | CAGACCTTCCCAGGGCACTCA |
| MICU1 | Forward | CTGAGGCCAATTAACTGC |
|  | Reverse | GGCTGTTGTCATACTTCTCATGG |

**2. Primers used in gene cloning**

| **Gene** | | **Primer pair** | | **Primer sequence** |
| --- | --- | --- | --- | --- |
| MCU | | Forward | | GCGGATCCCGTTTCCAGTTGAGAGATGGCGGCC |
|  |  | Reverse | | GCGAATTCGCCAGGATTCAGAGGCTTTTTGCAG |
| TFAM | | Forward | | GCGGATCCATGGCGTTTCTCCGAAGCATGT |
|  |  | Reverse | | GCGAATTCTTAACACTCCTCAGCACCATAT |
| **3. Primers for TFAM site-directed mutagenesis** | | | | |
| **Mutation site** | **Primer pair** | | **Primer sequence** | |
| S55A | Forward | | GTTGTCCAAAGAAACCTGTAGCTTCTTACCTTCGATTTTC | |
|  | Reverse | | GCTACAGGTTTCTTTGGACAACTTGCCAAGACAGATGA | |
| S160A | Forward | | CAAAAAGACCTCGTGCAGCTTATAACGTTTATGTAG | |
|  | Reverse | | CACGAGGTCTTTTTGGTTTTCCAAGCAGTG | |
| S177A | Forward | | GAAGCTAAGGGTGATGCACCGCAGGAAAAGCTG | |
|  | Reverse | | CATCACCCTTAGCTTCTTGGAATCTTTCAGC | |
| S55D | Forward | | GTCCAAAGAAACCTGTAGATTCTTACCTTCGA | |
|  | Reverse | | TCTACAGGTTTCTTTGGACAACTTGCCAAGAC | |
| S160D | Forward | | CCAAAAAGACCTCGTGACGCTTATAACGTTTATG | |
|  | Reverse | | GTCACGAGGTCTTTTTGGTTTTCCAAGCAG | |
| S177D | Forward | | GAAGCTAAGGGTGATGACCCGCAGGAAAAGC | |
|  | Reverse | | GTCATCACCCTTAGCTTCTTGGAATCTTTC | |

**4.siRNA**

| **siRNA** | **Primer pair** | **Primer sequence** |
| --- | --- | --- |
| siMCU | Sense | CUUCGACACUCAUGCCUUA |
|  | Anti-sense | UAAGGCAUGAGUGUCGAAG |
| siTFAM | Sense | ACAGGTTTCTTTGGACAACTTTTTTGGAAA |
|  | Anti-sense | TCTCTTGAAACAGGTTTCTTTGGACAACG |

**Supplemental table 3. Primary antibodies used for Western blotting and immunohistochemistry.**

| **Antibody** | **Company (Cat.NO.)** | **Working dilutions** |
| --- | --- | --- |
| MCU | SIGMA (HPA05189) | WB: 1/200; IHC 1/150 |
| β–actin | TDY BIOTEC (TDY051C) | WB: 1/2000 |
| UQCRC2 | Proteintech (14842-1-AP) | WB: 1/900 |
| MTCOX2 | Proteintech (55070-1-AP) | WB: 1/1000 |
| ND1 | Proteintech (19703-1-AP) | WB: 1/1000 |
| SDHB | Abcam (ab14714) | WB: 1/200 |
| ATP5A1 | Proteintech (14676-1-AP) | WB: 1/1000 |
| TFAM | Abcam (ab176558) | WB:1/1000; IP: 1/1000 |
| Anti-Phosphoserine | Abcam (ab9332) | WB: 1/1000 |
| Ki67 | MAIXIN-BIO (MAB-0542) | IHC:1/150 |
| COX4 | Cell Signaling (4844S) | IHC:1/2400 |
| NFκB p65(D14E12) XP | Cell Signaling (8242) | WB: 1/500 |
| Phospho- NFκB p65 | Cell Signaling (3031) | WB: 1/500 |
| DRP1 | Abcam (ab193216) | WB: 1/1000 |
| DRP1^S616^ | Cell Signaling (3455) | WB: 1/1000 |
| TOMM20 | Abcam (ab186734) | WB: 1/1000 |
| OPA1 | Abcam (ab157457) | WB: 1/1000 |
